# Point of use production of liposomal solubilised products

**DOI:** 10.1016/j.ijpharm.2017.12.012

**Published:** 2018-02-15

**Authors:** Swapnil Khadke, Peter Stone, Aleksey Rozhin, Jerome Kroonen, Yvonne Perrie

**Affiliations:** a Strathclyde Institute of Pharmacy and Biomedical Sciences, University of Strathclyde, Glasgow, Scotland G4 0RE, UK; b School of Life and Health Sciences, Aston University, Birmingham, B4 7ET, UK; c School of Engineering and Applied Science, Aston University, Birmingham, B4 7ET, UK; d Diagenode, Liege Science Park, 3 Rue bois Saint-Jean, 4102 Ougrée, Belgium

**Keywords:** Liposomes, Solubilisation, Low solubility drugs, Sonication, Personalised Formulation, Rapid screening

## Abstract

With the progression towards personalised and age-appropriate medicines, the production of drug loaded liposomes at the point of care would be highly desirable. In particular, liposomal solubilisation agents that can be produced rapidly and easily would provide a new option in personalised medicines. Such a process could also be used as a rapid tool for the formulation and pre-clinical screening of low soluble drugs. Within this paper, we outline a novel easy-to-use production method for point of use production of liposome solubilised drugs. Our results demonstrate that pre-formed multilamellar liposomes, stored in a fresh or frozen format, can be bilayer loaded with low solubility drugs using a simple bath sonication process. Sonication is undertaken in a sealed vial allowing the contents to remain sterile. Liposomes around 100 nm were prepared and these liposomes were able to increase the amount of drug dissolved by up to 10 fold. These liposomal solubilisation agents were stable in terms of size and drug solubilisation for up to 8 days when stored in the fridge making them an easy to use and robust small-scale tool for drug solubilisation.

## Graphical abstract

**Figure:**
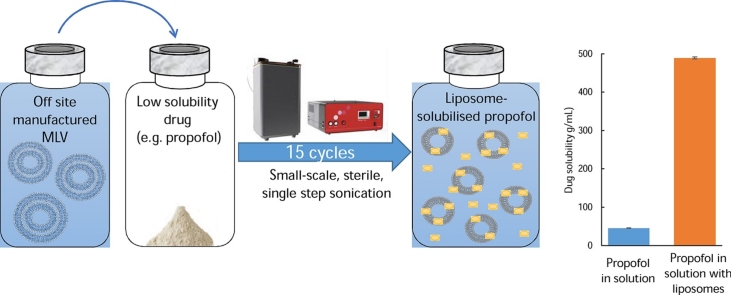

## 1 Introduction

Both the pre-clinical development and clinical use of many drugs remains hindered by their low solubility. Indeed, the ability to produce medicines in a liquid format remains a major consideration in pediatric and children’s medicines. Liquid dosage forms can also offer advantages as age-appropriate formulations, they offer flexibility in dosing and provide wider options for those who suffer from dysphagia. Tablets in particular can cause issues for pediatric dosing; for example, the World Health Organization noted that 4 children under 36 months in age died due to choking in a deworming campaign in Ethiopia during 2007 (WHO, 2007). They also noted that medical personnel are having to either break up tablets, dissolve them in solvents, or administer the powder contained in a capsule to young children as a relevant liquid drug delivery system isn’t available for that drug. However, there are a number of risks associated with these methods including difficulties in splitting and dividing of tablet doses and ensuring the drug can be reconstituted in water in a homogeneous system. Therefore, new solutions for such medicines are needed to overcome these issues.

Similar issues are faced with low solubility drugs in early pre-clinical development. Therefore a standard solubilizing agent that can be adopted for poorly soluble active pharmaceutical ingredients at a range of concentrations, that avoids the use of solvents, and that is non-toxic and easy to use would accelerate preclinical formulation time-lines. There are a number of different techniques used for formatting low solubility drugs, and suspension formulations are commonly used in the early discovery phase, owing to their ease of preparation. However, disadvantages associated with these systems can include batch-to-batch variability (including particle size) and stability issues.

Liposomes have been extensively investigated for the delivery of both hydrophobic and hydrophilic drugs due to their hydrophilic core and hydrophobic bilayer structure (Gregoriadis and Perrie, 2010). However, despite these advantages, their wide-scale use as clinically approved products remains limited to a small number of high-cost products: a key issue that has hindered their application is their cost-effective manufacture. As a result, the application of liposomes as solubilisation agents is generally cost-prohibitive. Yet liposomes offer the potential to act as solubilisation agents in a range of applications, including point-of-care medicine manipulation and point-of-use preclinical studies. For example, in early work from our group (Mohammed et al., 2004), we were able to load ibuprofen into the liposome bilayer and use liposomes as a lipophilic drug carrier. These studies identified key factors to consider in the formulation of liposomal solubilising agents. This included cholesterol bilayer content, lipid alkyl length and the presence of charged lipid head-groups (Mohammed et al., 2004). Following on from this, we considered a range of drugs (propofol, ibuprofen, phenytoin, diazepam and midazolam and rifampicin) and demonstrated that fatty alcohols could be used as bilayer stabilisers as an alternative to cholesterol (Ali et al., 2013). This work also showed that drug molecular weight is a key factor influencing drug loading within liposomes bilayers, with the larger molecules such as rifampicin showing low drug bilayer loading compared to smaller molecules such as propofol and ibuprofen (Ali et al., 2010; 2013). Kaess and Fahr (2014) has also shown that by taking advantage of the increased lipophilic relative area afforded to small liposomes compared to larger ones, they were able to load temoporphin into a number of different liposomal formulations.

Thus, whilst liposomes offer the potential to act as solubilisation agents, there remains a lack of appropriate, rapid and cost-effective production methods to allow the use of liposomes as solubilisation agents. Formation of large vesicles followed by size-reduction via sonication is a well-established method for the production of small-unilamellar liposomes in the laboratory setting. Yet this method is generally not suitable for the production of liposomes beyond the laboratory due to its multi-step process and lack of scalability. Sonication is a commonly used small-scale tool for size reduction and can be split into two options; bath and probe sonication. Probe sonication is the usual method employed due to its ease of use. It is also a well characterized and rapid method (e.g. Lapinski et al., 2007, Paini et al., 2015, Mendez and Banerjee, 2017). However, it can be limited by a lack of temperature control and the need to remove contamination post sonication (e.g. titanium particles that have sheared off the probe (Philippot et al., 1994)). Furthermore, this method cannot be conducted under sterile conditions due to the contact required between the sample and the probe. In contrast, bath sonication, can be conducted under sterile conditions (Lasic, 1998) and offers the opportunity to produce liposomes solubilizing drug at the individualized patient scale. It also offers the ability to work with low levels of active pharmaceutical ingredient, as is often the case in early formulation studies. Therefore, given that there is a need for the rapid and simple formulation of low solubility drugs, the aim of this current study was to develop a simple and rapid method for producing liposome solubilized drug formulations in a point of use setting. Our objectives were to investigate if drugs could be solubilized into pre-formed liposomes via sonication and the impact of drug and lipid selection had on this process.

## 2 Materials and methods

### 2.1 Materials

Ibuprofen, midazolam, propofol and cholesterol were purchased from Sigma-Aldrich, Dorset, UK. 1,2-distearoyl-sn-glycero-3-phosphocholine (DSPC), 1,2-distearoyl-sn-glycero-3-phospho-(1′-rac-glycerol) (DSPG), were obtained from Avanti Polar Lipids, Alabama. All chemicals used were of analytical grade and were used without further modification.

### 2.2 Methods

#### 2.2.1 Preparation of multilamellar vesicles

MLV were generated using a technique based on the established film method and modified for low solubility drugs. Briefly the lipid entities were dissolved in a chloroform:methanol (9:1) at appropriate ratios and the solvent evaporated on a rotary evaporator to yield a dry film as per the standard lipid film hydration method. To entrap drugs within the bilayer, the required amount of drug was added to the solvent mixture and subsequently hydrated. Liposomes were formed from DSPC:Chol; 4:1 M ratio, or DSPC:Chol:DSPG; 6:4:2.5 M ratio. In all cases, the film was hydrated with 2 mL of phosphate buffer saline (PBS) to give final lipid concentration of 2 mg/mL unless otherwise stated.

#### 2.2.2 Preparation of small unilamellar vesicles

**Fig. 1:**
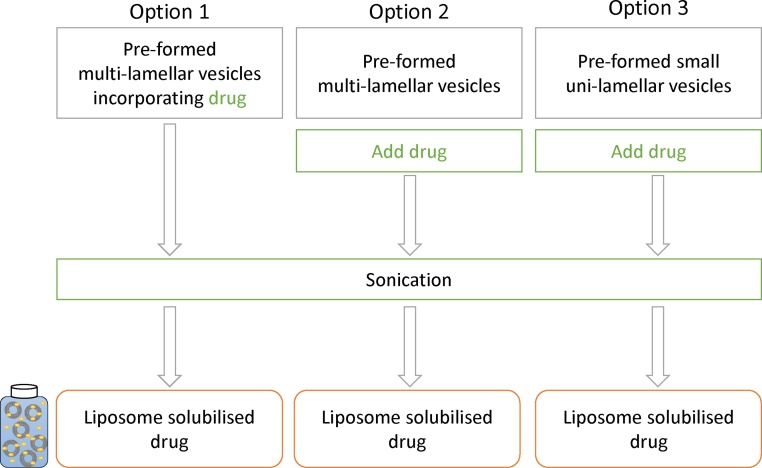
Method overview. Schematic representation of the three processes tested for promoting liposome-bilayer drug loading in a rapid small-scale format.

To prepare small unilamellar vesicles (SUV), 1 mL of MLV were subjected to sonication for 15 sonication cycles (90 s/cycles) with 30 s stop time between each sonication cycle using a Bioruptor® Plus sonicator at 40 °C. Using this system we are able to uniformly sonicates multiple samples (3–12 samples) of volume from 100 uL to 20 mL in sealed tubes. This system uses ultrasounds derived from magnets placed below the water tank and indirectly transfers ultrasonic energy to samples. The control of the temperature and the distribution of the energy inside the water bath and the continuous rotation of tubes promoted even sonication of samples. Each of the 3 different drugs were added independently to liposomes using 3 different options; option 1: drug was added during MLV preparation followed by sonication; option 2: drug was added post MLV formation but prior sonication; and option 3: drug was added to pre-made SUV and subjected to sonication (Fig. 1).

#### 2.2.3 Determination of drug loading in liposomes

Liposomes loaded with drug were subjected to dialysis against PBS for 3 h using dialysis membrane (MWCO: 12–14 kDa) to remove unloaded drug from liposomes. After dialysis, loaded liposomes were dissolved in methanol and analysed using UV-HPLC (Thermo Scientific, UK) to calculate drug loading at the appropriate wavelength for each compound (Ibuprofen, 221 nm; Midazolam, 258 nm and Propofol, 268 nm). A Luna 5 μ C18, 150 mm × 4.6 mm 100 A, phenomenex column was used to for HPLC separation using 0.1% TFA in water and methanol as mobile phase. Standard calibration curves of drug were used to calculate drug content in liposomes.

#### 2.2.4 Determination of particle size, polydispersity and zeta potential

The z-average diameter and polydispersity (PDI) of liposomes was determined by dynamic light scattering using the photon correlation spectroscopy (PCS) technique measured on a Malvern Zetasizer Nano-ZS (Malvern Instruments Ltd., UK). Using the cuvettes supplied by Malvern, 100 μL of the sample was diluted by the hydration phase (e.g. PBS buffer solution) up to 1 mL and the vesicle size and PDI was measured at 25 °C. The zeta potential of the liposomes was measured on Malvern Zetasizer Nano-ZS (Malvern Instruments Ltd., UK) at 25 °C. To measure the zeta potential 100 μL of liposome suspension was diluted in 900 μL of its aqueous phase (1:300 v/v PBS).

#### 2.2.5 Liposome stability studies

Liposomal size, PDI, zeta potential and drug retention were used as parameters to indicate the physical stability of liposomes. The stability of formulations, with respect to retention of the entrapped drug and changes in the size distribution, were determined by incubating vesicles (after separation of the free drug) in PBS at 4–8 °C. Initially, three independent samples of drug loaded liposomes were prepared for each time intervals. Immediately after preparation, and at time intervals of 24, 48, 96 and 192 h, samples were analysed.

## 3 Results

### 3.1 Sonication of pre-formed MLV in the presence of drug can promote effective drug solubilisation in a sterile and easy to use method

The objective of this work was to investigate three different methods for the loading of lipophilic drugs using the bath sonicator, as outlined in Fig. 1. Three options were considered; option 1 involves the traditional lipid film hydration method with the drug added at the initial stage of the lipid film formation. This method is generally restricted to a laboratory setting. In Option 2 the drug is mixed with preformed multilamellar vesicles (MLV) and subjected to sonication. This would allow pre-formed MLV to be prepared within appropriate manufacturing facilities and then a rapid 1 step-process to convert these liposomes into drug loaded small unilamellar vesicles (SUV). In option 3, the drug is mixed with preformed SUV and subject to sonication to promote drug solubilisation. Our liposomes were formed from DSPC and cholesterol based on previous studies we had undertaken with liposomes as drug solubilizing agents, where we show that longer alkyl chain length lipids promote higher bilayer loading (Mohammed et al., 2004). Cholesterol content was also shown to impact on bilayer loading, with the presence of cholesterol promoting liposome stability yet hindering bilayer drug loading and therefore a DSPC:Chol lipid weight ratio of 10:4 was selected to meet the needs of both good liposome stability and bilayer drug loading.

#### 3.1.1 Sonication protocol optimization

**Fig. 2:**
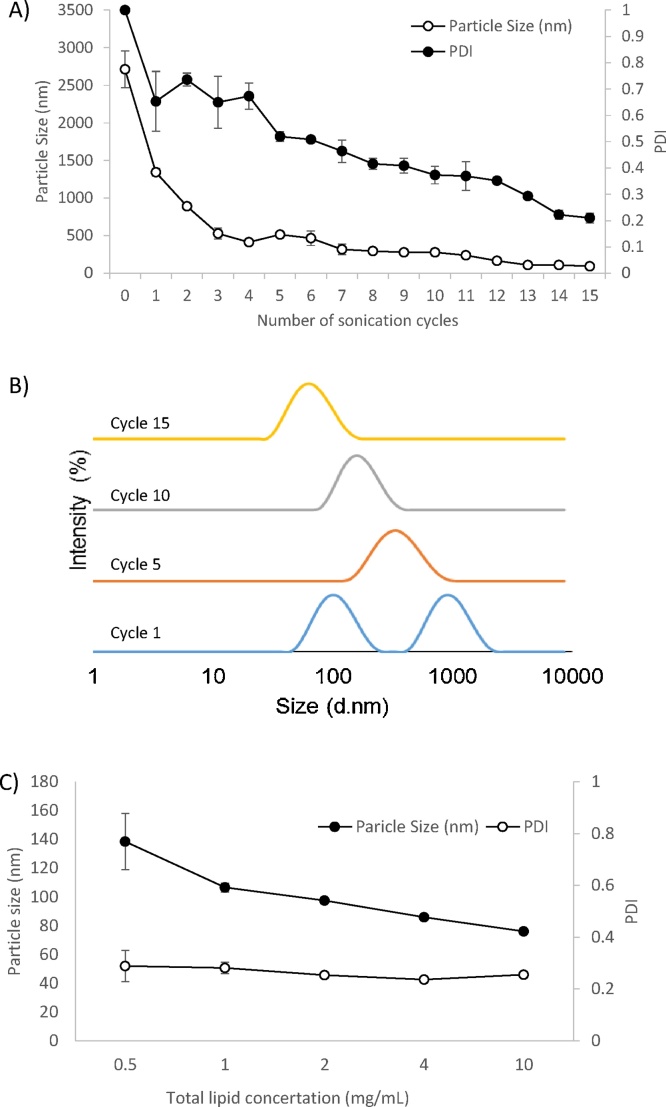
The effect of sonication cycles on liposome particle size attributes. Liposome (DSPC:Chol; 4:1 M ratio, 2 mg/mL) size and polydispersity (A) and intensity plots (B) of vesicles subjected to 15 sonication cycles (90 ss/cycle). The effect of lipid concentration on particle size and PDI (C) was also measured. Results represent mean ± SD, for 3 independent experiments.

To develop a rapid point-of-use process for the production of liposome-solubilised drug, initially the sonication process was optimised (Fig. 2). Using 15 sonication cycles of 90 ss/cycle produced liposomes 100–120 nm in size with PDI of 0.2 and a single particle size population were prepared (Fig. 2A and B). The effect of lipid concentration on particle size reduction was also investigated (Fig. 2C). Lipid concentrations between 1 and 10 mg/mL were all effectively reduced to vesicle sizes between 100 and 120 nm with PDI of 0.2 demonstrating that a wide range of lipid concentrations could be effectively size reduced using the same sonication protocol. Lower lipid concentrations (0.5 mg/mL) tended to give slightly higher and more variable size ranges (120–160 nm) but again with a PDI range of 0.2 (Fig. 2C).

#### 3.1.2 Selection of drug loading process

**Fig. 3:**
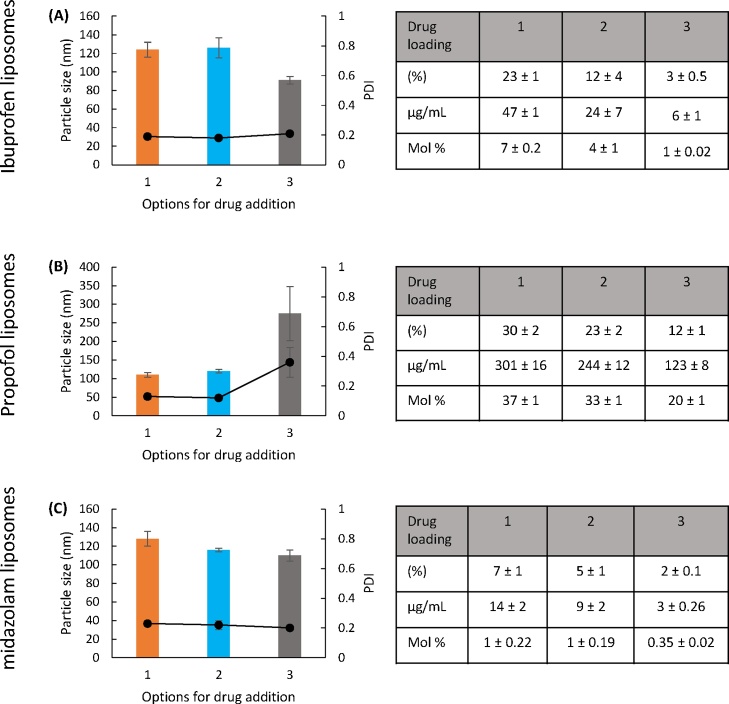
Drug bilayer loading via sonication. Three methods of formulating liposomes (DSPC:Chol; 4:1 M ratio, 2 mg/mL) were tested; liposomes were prepared by lipid hydration and drug loading was achieved as outlined in Fig. 1. Particle size, PDI and drug loading via the three different methods for ibuprofen, propofol and midazolam are shown in A, B and C respectively. Results represent mean ± SD, for 3 independent experiments.

To consider drug loading, three options were considered as outlined in Fig. 1. In all three options, the liposomes were sonicated for 15 cycles as shown in Fig. 2. As can be seen in Fig. 3, both option 1 and 2 gave similar vesicle sizes and PDI across the three drugs tested (particle sizes 100–140 nm, PDI 0.2; Fig. 3) with neither the point of addition of the drug into the liposomes nor the drug loaded having an impact. However, in the case of pre-formed SUV mixed with drug and subjected to sonication, there was some variability in size and PDI particularly in the case of propofol with sizes increasing to 250 nm (Fig. 3B) which may be a results of over sonication and stress damage to the lipids which can promote aggregation.

In terms of drug loading within the liposomes, results in Fig. 3 are presented as % drug loading, drug loading as μg/mL, and as mol% as drug solubilisation is reported in these various ways. The results in Fig. 3 show that for all three drugs, addition of the drug during the formation of MLV (option 1) gives the highest drug loading (23%, 30% and 7% for ibuprofen, propofol and midazolam; Fig. 3A–C respectively). Mixing of drug with pre-formed MLV followed by sonication gives the second highest level of drug loading (option 2; 12%, 23% and 5% for ibuprofen, propofol and midazolam respectively; Fig. 3). Finally option 3 (mixing drug with pre-formed SUV followed by sonication) is the least efficient method for promoting bilayer loading (3%, 12% and 2% for ibuprofen, propofol and midazolam respectively; Fig. 3). When comparing between the drugs, propofol was shown to give the highest drug loading in all three options, in line with our previous studies using MLV (Ali et al., 2013).

**Fig. 4:**
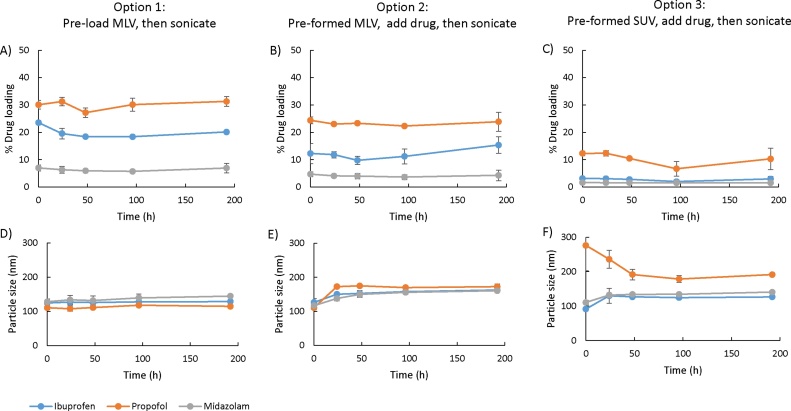
Short-term stability of liposomes. Liposomes (DSPC:Chol; 4:1 M ratio, 2 mg/mL) were prepared and loaded with either ibuprofen, propofol or midazolam by the three methods (option 1: A and D; option 2: B and E; option 3: C and F) and stored at 2–8 °C. Drug loading (A, B, C) and particle size (C, D, E) were measured at time intervals. Results represent mean ± SD, for 3 independent experiments.

To consider the stability of the liposome-solubilized drug systems, liposomes prepared by all three loading methods, with the three drugs loaded, were subjected to a short-term stability study. Liposomes were stored at 2–8 °C for up to 192 h to investigate the potential of storing these systems in the fridge in line with extemporaneously prepared oral suspensions which can be stored for 1 week. The results in Fig. 4 demonstrate that drug-loaded liposomes formulated by option 1 or 2 were stable over the test period, maintaining their size and retaining their drug loading for all three drugs tested. When pre-loaded SUV were sonication-loaded with drug, there was some initial variability in vesicle size and reductions in the already low drug loading were noted over time. From the results in Fig. 3, Fig. 4, option 1 gave the highest drug loading; however, option 2 offers both appropriate drug loading in combination with an easy to use process that could be adopted in a range of environments using pre-manufactured MLV solutions.

#### 3.1.3 Liposome solubilisation capacity

**Fig. 5:**
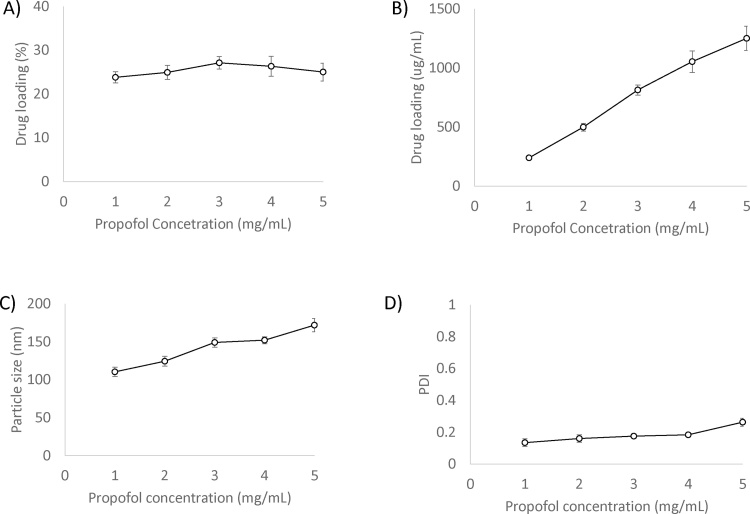
Effect of initial drug concentration in terms of propofol loading (% loading (A), μg/mL (B)), particle size (C) and PDI (D). Liposomes composed of (DSPC:Chol; 4:1 M ratio, 2 mg/mL) were prepared with increasing propofol concentrations from 1–5 mg/mL. Results represent mean ± SD, for 3 independent experiments.

To consider the solubilisation capacity, liposomes at a concentration of 2 mg/mL (total lipid) were loaded with propofol over a range of concentration (1–5 mg/mL) using option 2. The results in Fig. 5 demonstrate that % drug loading remained at 23–25% across the concentrations used and resulted in absolute drug loading increasing up to 1250 ug/mL (Fig. 5A and B respectively). However, at increasing propofol concentrations, vesicle size was shown to increase from 110 to 170 nm, with a corresponding increase in PDI to 0.26 at 5 mg/mL propofol (Fig. 5C and D). Therefore depending on the application, the lipid:drug ratio should be considered, as this is often a key critical attribute of a liposomal formulation.

### 3.2 Liposomal-solubilised drugs can be rapidly prepared using pre-formed and frozen liposomes

**Fig. 6:**
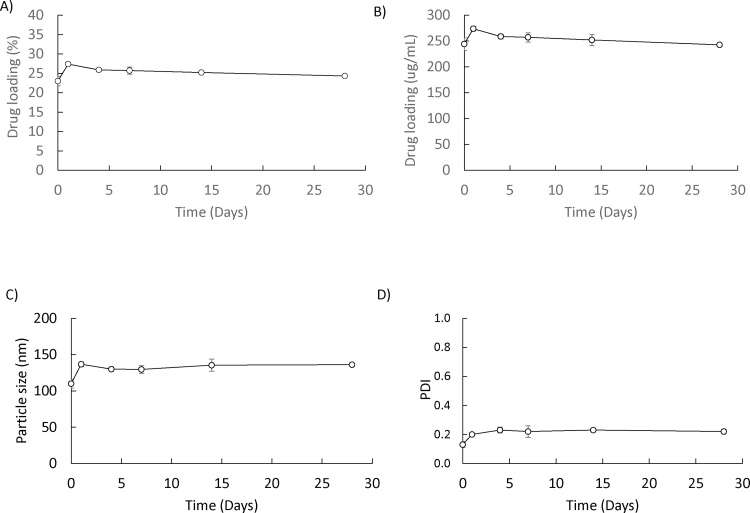
Stability of liposomal-drug formulations prepared from frozen MLV. MLVs were stored at −20 °C for 1, 4, 7, 18 and 28 days. After thawing, drug was added via option 2 (Fig. 1) with liposomes sonicated for 15 sonication cycles (90 ss/cycle). Drug loading (A and B), particle size (C) and PDI (D) is shown respectively. Results represent mean ± SD, for 3 independent experiments.

To consider the potential of using pre-manufactured MLV stored in a frozen format (thereby potentially increasing their long-term storage stability), a batch of MLV liposomes prepared at the usual 2 mg/mL were prepared, and stored frozen for up to 28 days. These liposomes were then defrosted and used to prepare liposomal solubilisation as per option 2. The results show that frozen MLV stocks were as effective as freshly prepared MLV in terms of propofol loading and vesicle size (Fig. 6), demonstrating that pre-manufactured liposomes could be shipped/stored in a frozen format prior to use to extend their long term stability Table 1.

**Table 1:** Attributes of drugs solubilised within liposomes.

Drug	Structure	Molecular weight (Da)	Water solubility mg/mL at 25 °C	log P	pK_a_
Propofol		178	0.124	3.79	11.1
Ibuprofen		206	0.021	3.97	4.9
Midazolam		326	0.024	4.33	5.5

### 3.3 Incorporation of anionic lipids enhances drug solubilisation capacity

**Fig. 7:**
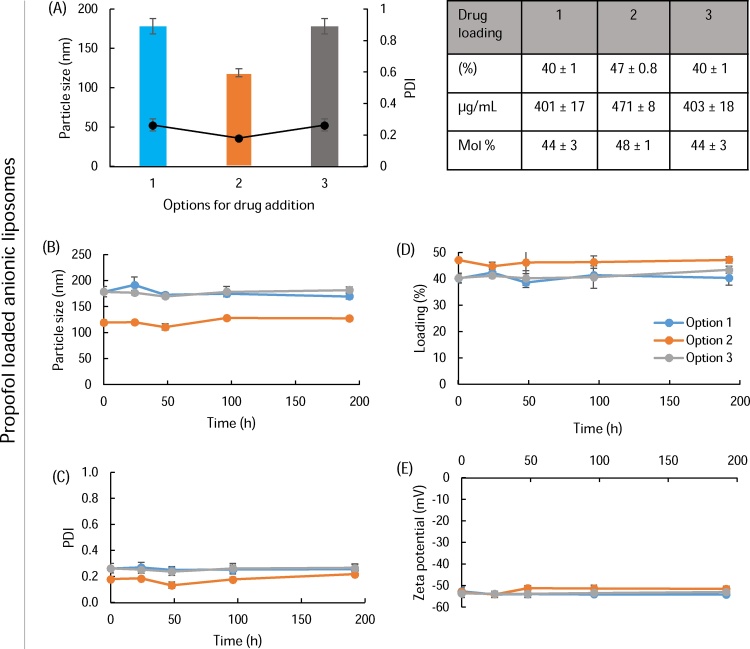
Propofol loaded into anionic liposome formulations. Three methods of formulating liposomes (DSPC:Chol:DSPG; 6:4:2.5 M ratio, 2 mg/mL) were tested. Particle size, PDI and drug loading via the three different methods for propofol are shown in A. Short term stability at 2–8° for up to 192 h via the three different methods for propofol was also measured and particle size (B), PDI (C), drug loading (D) and zeta potential (E) are shown respectively. Results represent mean ± SD, for 3 independent experiments.

The liposomes used in Fig. 2, Fig. 3, Fig. 4, Fig. 5, Fig. 6 were formulated from a simple DSPC:Cholesterol (4:1 M ratio) formulation. This was based on early studies demonstrating that low levels of cholesterol were able to stabilize the vesicles without inhibiting bilayer drug loading (Mohammed et al., 2004). However, the addition of charged lipids is commonly used to enhance liposome stability and may impact on bilayer drug loading. Therefore, we also investigated an anionic liposome formulation incorporating DSPG, as PG lipids are used within several commercially available liposome products. Following sonication, empty liposomes were 100–120 nm in size, similar to DSPC:Chol liposomes. In terms of propofol drug loading, this was higher for the anionic liposome formulation for all three methods of manufacture (Fig. 7A) when compared to DSPC:Chol liposomes (Fig. 2) with the anionic liposomes giving 40% propofol loading (1 mg/mL initial amount added). These liposomes were stable over 8 days, similar to the neutral DSPC:Chol liposomes (Fig. 7B–E). Again these results show that loading drug within pre-formed MLV is a rapid and convenient option for producing liposomal-solubilised drug. Similarly, pre-formed and frozen MLV can be used in this process to produce liposome-solubilised drug rapidly (Table 2) which offers an easy way to store such pre-formed liposomes for longer periods. This also offers the options of larger batches of MLV to be prepared, aliquoted and stored ready for use.

**Table 2:** Drug solubilisation within freshly prepared and frozen anionic liposomes. Anionic MLV (DSPC:Chol:DSPG; 6:4:2.5 M ratio, 2 mg/mL) were stored at −20 °C for 24 h. After thawing, drug was added via sonicated for 15 sonication cycles (90 ss/cycle). Results represent mean ± SD, for 3 independent experiments.

**Initial stocks**	**Physicochemical characterisation of resultant SUV**			**Propofol loading**		
	**Size (nm)**	**PDI**	**ZP (mV)**	**% loading**	**μg/mL**	**Mol%**
Freshly prepared MLV	138.5 ± 2.3	0.16 ± 0.01	−52.7 ± 4.2	40 ± 1	408 ± 17	51 ± 0.2
Frozen MLV	126.3 ± 6.5	0.19 ± 0.01	−54.0 ± 8.6	47 ± 1	470 ± 7	48 ± 0.4

**Fig. 8:**
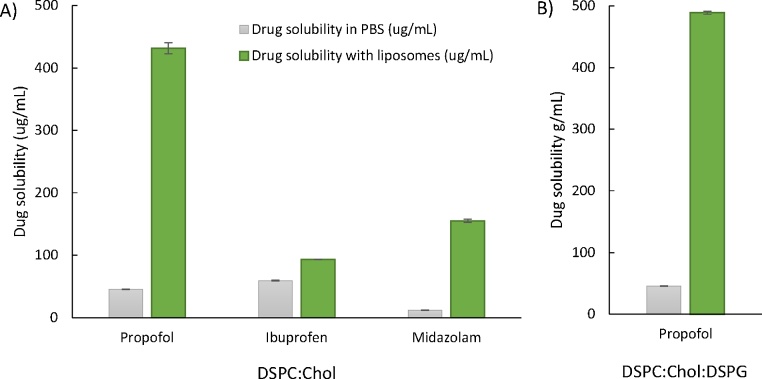
Comparison of drug solubility with and without A) neutral liposomes and B) anionic liposomes present. Drug was subjected to sonication for 15 sonication cycles (90 ss/cycle) with or without liposomes and centrifuged for 1 h at 20,000 g and filtered through 0.22 um syringe filter. Results represent mean ± SD, for 3 independent experiments.

In Fig. 3, Fig. 4, Fig. 5, Fig. 6, Fig. 7, the results are shown as drug solubilized within the liposome bilayers (with non-entrapped drug being removed). This would be appropriate for drug delivery options where the bilayer-loaded drug distribution is to be dictated by the liposomes, as in the case of Ambisome® for example. Liposomes are well recognized for their ability to passively target either the mononuclear phagocytic system, sites of inflammation and/or tumour sites depending on their structural design (Gregoriadis and Perrie, 2010). When considering their capacity as solubilizing agents, where it is total drug dissolved that is the key, the overall enhanced solubility offered by employing liposomes is shown in Fig. 8. Here we show the total amount of drug in solution (i.e. free dissolved drug is not removed from the liposome suspension); both the DSPC:Chol and DSPC:Chol:DSPG liposomes offer around a 10 fold increase of drug in solution (Fig. 8).

## 4 Discussion

There are many options available to improve the solubility of drugs including pH and salt forms, co-solvents and micelle solubilisation, solid dispersions and co-crystals, inclusion complexation, emulsification and nanotechnologies. Each of these systems offers a range of advantages and disadvantages. In the case of liposomes, they offer the ability to improve solubility without the use of co-solvents and are stable upon dilution. Liposomes can also protect the drug from degradation and can be designed to dictate the drug biodistribution. Liposomes can be produced using lipids that are relatively low cost and they have a proven track-record clinically. However, the use of liposomes is limited by their complex manufacturing processes. In many of these methods we rely on size reduction of large vesicles. In the laboratory setting this normally starts with the lipid film hydration method. To reduce vesicle sizes, a range of methods can be adopted including sonication, shear or pressure forces, including microfluidization, high-pressure homogenization or other shear force-induced homogenizer to reduce vesicle size (Wagner and Vorauer-Uhl, 2011, Szoka and Papahadjopoulos, 1980).

To support their more accessible use and provide a rapid low-cost solubilisation tool, we have developed a novel and simple small-scale drug loading method has been developed. Sonication is a well-recognized tool for size reduction of liposomes with some of the first liposomal studies employing this method (e.g. Papahadjopoulos and Miller, 1967, Papahadjopoulos and Watkins, 1967). Probe sonication is a cheap, simple and heavily used method but is limited by potential lipid degradation resulting from overheating (Uchegbu, 2013, Lapinski et al., 2007). In contrast, bath sonication is a non-contact option that can be temperature controlled. Previously studies by Lapinski et al., 2007 using rotational and translational diffusion of an embedded chromophore have shown that liposomes formed by bath sonication and extrusion exhibit the same molecular scale environment despite differences in size. Indeed, the molecular scale organization is determined by lipid interactions. Therefore, there is the potential that a similar protocol exploiting extrusion, rather than sonication, could achieve similar results. However, extrusion on a small scale can be laborious and is more cost effective at larger scales. Therefore, the potential of the low cost bath sonication protocol to maintain a closed sterile formulation under controlled temperature conditions and have a set programme with minimal user input offers key advantages.

Obviously, in addition to their ability to improve drug solubility, liposomes can be used to control the delivery of drugs, with around 15 liposome products currently licensed for this use. With many of these products, active loading is used for optimized loading of ionizable drugs and this is measured by drug-to-lipid ratios as this ratio often impacts on stability and drug release rates (Modi et al., 2012). However, the extremely low aqueous solubilities of many drug candidates can limit the external driving force, thus slowing liposomal uptake during active loading (Modi et al., 2012) and therefore can limit its application in small scale batches with low solubility drugs. Whilst the above bath-sonication platform technology can also be applied for drug delivery systems, this method is not appropriate for aqueous soluble drugs and an additional step to remove non-incorporated drug is required if considered for in vivo use parenterally. There are also the various quality control and quality assurance aspects that would need to be considered for point-of-care production of any medicine. However, for bilayer loaded drugs, this method can be used for not only enhance solubility but can enhance potency. This can make it a useful tool for pre-clinical studies. For example, work by Pandelidou et al (2011) demonstrated that the incorporation of curcumin within eff PC liposomes with high efficiency (85% when at a drug to lipid molar ratio of 1:14) and that the cytotoxicity against colorectal cancer cell lines was enhanced.

## 5 Conclusions

These studies were designed to challenge the dogma that liposomes cannot be easily prepared in a point-of-use setting. Here we show that liposomes could be manufactured and shipped/stored as multi-lamellar vesicles, then low-solubility drugs loaded into these liposomes at the point of use to enhance solubility using a rapid and easy to use single-sonication step. Whilst bath-sonication is a commonly used method, its use as a single step drug loading method has thus far not been reported. This new protocol can easily be adopted in pre-clinical studies in vitro and in vivo (with an additional non-incorporated drug removal step if administrated parenterally). This is particularly useful when API amounts are often limited. This protocol also offers the potential of preparing personalized liquid dosage formats which can overcome issues related to solid dosage forms.
